# Coronavirus Disease Health Care Delivery Impact on African Americans

**DOI:** 10.1017/dmp.2020.179

**Published:** 2020-05-29

**Authors:** Rahul Chaturvedi, Rodney A Gabriel

**Affiliations:** School of Medicine, University of California San Diego, La Jolla, CA; Department of Anesthesiology, University of California San Diego, La Jolla, CA; Department of Medicine, Division of Biomedical Informatics, University of California, San Diego, La Jolla, CA

**Keywords:** delivery of health care, health impact assessment, infectious disease medicine, mortality, public health

## Abstract

The severe acute respiratory syndrome coronavirus 2 (SARS-CoV-2) has infected over 1.5 million individuals and led to over 91, 000 deaths in the United States (US) alone as of May 20th, 2020. Minority populations, however, continue to be a high-risk population to contract the SARS-CoV-2 infection. While socioeconomic inequality may help to explain why minority ethnic populations are contracting the SARS-CoV-2 in larger proportions, the reason for elevated mortality rates in African Americans is still unknown. African Americans are less likely than whites to utilize high-quality hospitals, ambulatory care services, and regular primary care providers; this is most likely a result of barriers to accessing high quality treatment, as African Americans have substantially higher uninsured rates. However, previous reports have shown that regardless of insurance status, African Americans are more likely to be directed toward lower quality treatment plans compared to their white counterparts, and that physicians carry implicit biases that negatively impact treatment regimens for these minority populations. While income, education, and access to healthcare should be revised in due time, in the short term physicians should do everything possible to learn about implicit biases that may exist in healthcare, as the first step to minimize implicit biases is to recognize that they exist.

The severe acute respiratory syndrome coronavirus 2 (SARS-CoV-2) has infected over 1.5 million individuals and led to over 91 000 deaths in the United States alone as of May 20, 2020. Government policies have rightfully adopted the 6-feet physical and social distancing policies that have already begun to show improvements in intensive care unit admissions throughout the country. However, more and more news articles have been pointing out ethnic disparities in the epidemiology of coronavirus disease (COVID-19).^1-3^ So why is this the case? Certain minority groups, such as African-Americans, live in extremely dense housing and more frequently use public transportation, and thereby may not be able to reasonably abide by these social distancing policies.^4^ Low-income apartments have decreased distances between units, communal laundry services, and less frequent maintenance and grounds-keeping services. Ethnic minorities may also be overrepresented in public housing, homeless shelters, and prisons.^5,6^ Minority populations, thereby, continue to be a high-risk population to contract the SARS-CoV-2 infection. While socioeconomic inequality may help explain why minority ethnic populations are contracting the SARS-CoV-2 in larger proportions, the reason for elevated mortality rates in African Americans is still unknown. Theoretically, all patients who seek medical care should obtain the same quality of health care treatment. Unfortunately, the United States is far from adequately bridging the gap of ethnic disparities in health care delivery, and the SARS-CoV-2 epidemic has made this ever so apparent.

Fortunately, hospitals have begun collecting ethnicity data from patients with SARS-CoV-2. The lack of data collection thus far may be a result of the data being considered too sensitive or irrelevant, the lack of established policies for collecting the data, and inadequate incentives, as health care payers such as insurance companies do not mandate the data collection.^7^ Results have shown that, in Louisiana and Chicago, African Americans have consistently had at least a 30% higher total COVID-19-related death count as compared with their white counterparts. As per the daily COVID-19 data released by public health officials in New York City, the rate of COVID-19 deaths per 100 000 for African Americans has been around double that of whites throughout this crisis.^8-10^

The SARS-CoV-2 epidemic has thus reiterated a glaring issue in the health care industry – ethnic and racial disparities in health care are impacting mortality rates.^11^ The “Iron Triangle” of health care has long been known to comprise 3 essential components: access, cost, and quality.^12^ It is a well-known fact that all 3 cannot be optimized simultaneously, but with minority groups, we may not be improving on any of the components adequately. African Americans are less likely than whites to use high-quality hospitals, ambulatory care services, and regular primary care providers; this is most likely a result of barriers to accessing high-quality treatment,^13^ as African Americans and Hispanics have substantially higher uninsured rates.^14^ However, for those who do have access and for those who approach the emergency rooms during this frantic time for concerns of SARS-CoV-2, can we truly say that the quality of care for minority groups will be equal? Previous reports have shown that regardless of insurance status, African Americans are more likely to be directed toward lower quality treatment plans compared with their white counterparts, and that physicians carry implicit biases that negatively impact treatment regimens for these minority populations.^13,15-17^ Studies have also shown that physicians may perceive African Americans as less willing to adhere to medical advice, which may contribute to poor communication between white physicians and African American patients.^18,19^ These biases, in addition to the longstanding history of unethical experimentation on minority groups in the United States, lead to mistrust by African Americans and other minority groups and contribute to health care avoidance.^20,21^ This is particularly worrisome, as mistrust of the health care system by African Americans and other minority groups may lead to their avoidance of critical public health interventions, such as a future SARS-CoV-2 vaccine. The consequence of these ethnic disparities not only affects mortality rates, as we are seeing with this SARS-CoV-2 outbreak, but also impacts finances; reducing health disparities for minorities may have reduced direct medical expenditures by US $230 billion between the years 2003 and 2006.^22^

Numerous sources, including the Centers for Disease Control and Prevention, note that health care inequalities during this COVID-19 pandemic are due to factors such as living conditions, work circumstances, and access to health care.^23^ During the US H1N1 influenza pandemic in 2009, minority populations were more likely to live in metro areas, had more difficulty avoiding public transportation, and had more difficulty accessing day care for their children that was separate from others.^24^ The increased mortality rate per 100 000 in African Americans in New York City is certainly partially attributable to the above socioeconomic-related risk factors, such as the inability to maintain social distance in dense housing and various additional health comorbidities. African Americans have an increased prevalence of cardiovascular disease, including heart failure and cardiomyopathies, which are also poor prognostic indicators for COVID-19 patients.^25,26^ However, in states such as Alabama, we see a different picture – significantly more whites are being infected with SARS-CoV-2, but African Americans continue to comprise a similar, if not higher, percentage of total SARS-CoV-2-associated deaths.^27^ In fact, even though fewer African Americans are being infected with SARS-CoV-2 in Alabama, a higher mortality rate exists for African Americans with no underlying conditions. Implicit physician biases may contribute to these findings. One recent report from the biotech data firm Rubix Life Sciences noted that, in several states, African Americans with COVID-19 symptoms, such as cough and fever, were less likely to be referred for coronavirus testing.^28^

While the previous data should be enough to spur more research on the topic, it only encompasses less than 25% of cases, as hospitals have not gathered ethnicity information for all patients. The etiology of this health care inequity is complex, as survival in the pandemic relies on income, education, and access to health care. However, as described previously, there are implicit biases that physicians harbor that may be contributing to health care delivery inequality as well. The SARS-CoV-2 epidemic has shown us how important it is to address current health care inequity. While income, education, and access to health care should be revised in due time, in the short term, physicians should do everything possible to learn about implicit biases that may exist in health care, as the first step to minimize implicit biases is to recognize that they exist. Failure to recognize these biases during the COVID-19 pandemic will lead to increased morbidity and mortality of African Americans, as physicians may underutilize testing resources and African Americans may avoid health care personnel altogether due to mistrust. When a SARS-CoV-2 vaccine does become available, the biases and mistrust will lead to underutilization of a precious public health resource and thereby may contribute to the preventable spread of the virus and increased mortality among all ethnicities.
